# Supplementary material for: Surgeons versus computer vision: a comparative analysis on surgical phase recognition capabilities
Source: Int J Comput Assist Radiol Surg. 2025 May 31;20(6):1283–91. doi: 10.1007/s11548-025-03383-4 (PMC12167303; doi:10.1007/s11548-025-03383-4)
Supplement: Supplementary file 1 — (pdf 7431 KB) [file 11548_2025_3383_MOESM1_ESM.pdf]

Surgeons vs. Computer Vision: A comparative analysis on surgical phase recognition capabilities

Marco Mezzina[1,2*], Pieter De Backer[1,3], Tom Vercauteren[4], Matthew Blaschko[2], Alexandre Mottrie[1,3], Tinne Tuytelaars[2]

[1*]Orsi Academy, Belgium.
[2]Faculty of Engineering Science, KU Leuven, Belgium.
[3]Department of Urology, OLV Aalst Hospital, Belgium.
[4]School of BMEIS, King's College London, UK.

*Corresponding author(s). E-mail(s): marco.mezzina@orsi.be;

Supplementary Material - Tables

Table S1: Surgical phases description for RAPN surgery provided to participants at the start of the study. The textual description is the result of validated studies resulted in a protocol developed by surgeons for surgeons.

Phase	Description
**Port Insertion and Surgical Access**	The endoscopic camera captures the abdominal cavity. Trocars are being placed, instruments are introduced into trocars, and adhesion removal is performed.
**Colon Mobilization**	Identification of the mesentric line and incision of line of Toldt.
**General Hilar Control**	Identification, dissection and isolation of the main renal artery. A vessel loop is placed around the main renal artery.
**Selective Hilar Control**	Identification, dissection and isolation of secondary renal arteries. Vessel loops are placed around the secondary renal arteries.

Phase	Description
**Kidney Mobilization**	Incisions of Gerota's fascia to access the kidney. The kidney surface is freed from perinephric fat.
**Tumor Identification**	Localisation and delineation of the tumor. The ultrasound probe can be employed for localisation, while coagulation can be used for tumor marking.
**Hilar Clamping**	Bulldog clamp clamps the main renal artery and/or secondary arteries.
**Tumor Excision**	Resection of the tumor by separation from the renal parenchyma. Not to be confused with Tumor Identification.
**Specimen Retrieval and Deposition**	The tumor mass is placed to a convenient location for later retrieval.
**Inner Renorrhaphy**	Suturing of the inner layers of the kidney including running medullary sutures and repair of the urinary collecting system.
**Hilar Unclamping**	The bulldog clamp is removed from the main renal artery and/or secondary arteries.
**Outer Renorrhaphy**	Suturing/approximation of the kidney cortex. Non-absorbable clips are repeatedly attached to the ends of the suture wire to perform the sliding Hem-o-lok technique.
**Specimen Removal**	The tumor is placed inside an endobag for removal from the patient's body.
**Retroperitonealization of The Kidney**	The peritoneum is closed by performing parietal peritoneum suturing.
**Instrument Removal**	Vessel loops, bulldog clamps, robotic instruments, suture wires are removed from the patient's abdomen.

Table S2: Summary of user feedback per user group on the overall survey experience. The table includes only responses from participants who provided it.

**Medical Students**

- Very straightforward survey, well done and good luck!
- Too many unclear clips and images due to gauze/hemostatics, making phase recognition difficult.
- Impossible to answer questions with frames; some videos were not representative of phases.
- Some videos only showed needle movement, making identification hard.
- Suggested adding surgical anatomical videos for recognizing structures in robotic surgery.
- Difficult survey. Some videos don't represent certain phases and they should last several seconds. However, it's an interesting survey and it's not possible define how many seconds the videos should last.
- Pretty hard for medical students.
- Nice survey, but videos had desaturated colors.
- Videos should be longer, but defining the right length is tricky.
- Single image of clamp didn't clarify clamping/unclamping.
- Difficult frames and short videos.
- Even with short videos, distinguishing phases was possible, but frames alone were insufficient.
- Videos made it easier to answer.
- Some snippets weren't representative of the phases.
- Some phases, like hilar clamping/unclamping, were difficult to recognize, and no videos represented these phases.
- Difficult survey, but video snippets made answering easier.

Residents

- Very interesting exercise.
- A brilliant way to test and improve knowledge for residents.
- Suggested longer videos and avoiding non-steady frames or clips with no specific movement.
- Requested feedback on correct answers.
- Some questions were problematic: Question 8 had no correct answer (hemostasis phase); Question 12 had no video.
- Sometimes pictures alone were misleading and could represent multiple situations.
- Very difficult!

Fellows

- Difficult to match some steps with videos. For example, removing the vessel loop: Is it unclamping or instrument removal? Another example: showing the suture surface—is it outdoor suturing or unclamping? It seems like checking the suture after unclamping.

Consultants

- Suggested adding more detailed descriptions for some steps.
- The video should not be longer but should capture more relevant moments of the phases.
- Very interesting project.
- Not all phases shown were listed in the steps.
- Some videos showed "in between phases," potentially confusing both humans and AI.
- Hemostatic agents considered as outer renorraphy? Unclear.
- Some phases were "in between," making answers difficult at times.
- Some videos didn't show the specific part of the case in question.
- Would like the study's purpose better explained.

Table S3: Cholec80 test set performance reported as mean ± standard deviation. For the 40/40 split, the standard deviation is computed across all test videos, while for the 40/8/32 split, it is computed across multiple runs.

Metric	Model	Dataset Split (train/val/test)	
		40/40/0	40/8/32
**Accuracy (%)**	ResNet50+LSTM[?]	85.3±7.3	N/A
	ResNet50+LSTM (Our)	82.4±8.1	N/A
	TeCNO[?]	N/A	**88.6±0.3**
	TeCNO (Our)	N/A	84.9±0.4
**F1-Score (%)**	ResNet50+LSTM[?]	82.1±7.2	N/A
	ResNet50+LSTM (Our)	80.2 ± 7.6	N/A
	TeCNO[?]	N/A	**83.4±0.6**
	TeCNO (Our)	N/A	81.0±0.7

Supplementary Material - Figures

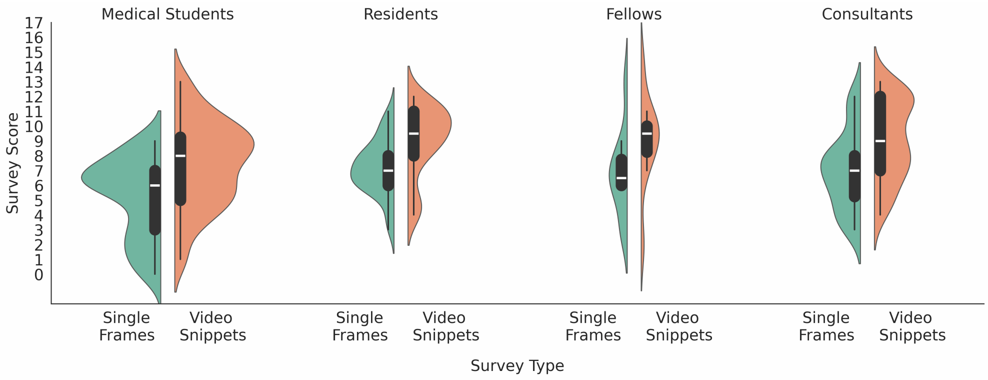

Fig. S1: Scores across professions, with Surgical Trainees divided into the original categories of Residents and Fellows. Densities are proportional to the number of observations.

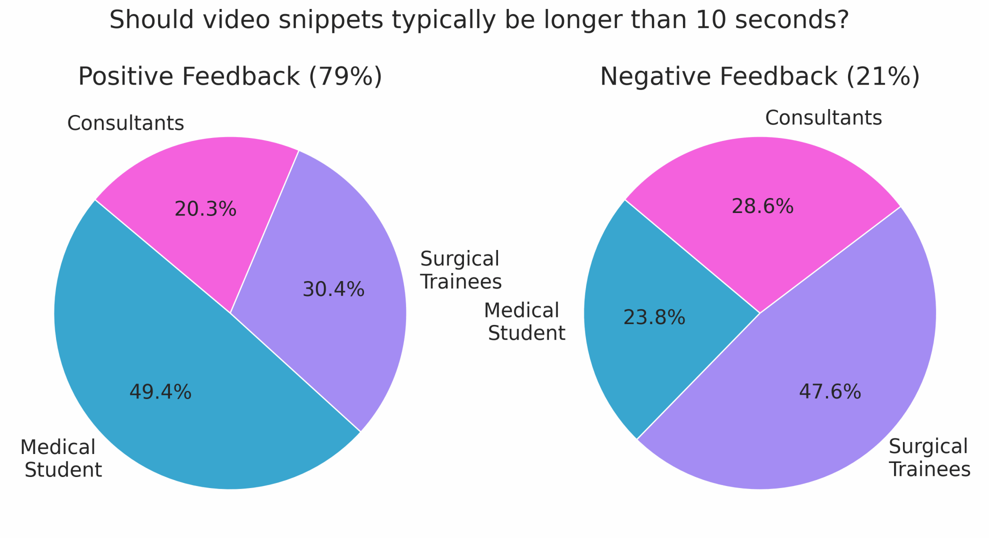

Fig. S2: Users feedback on the length of video snippets to solve the classification task.

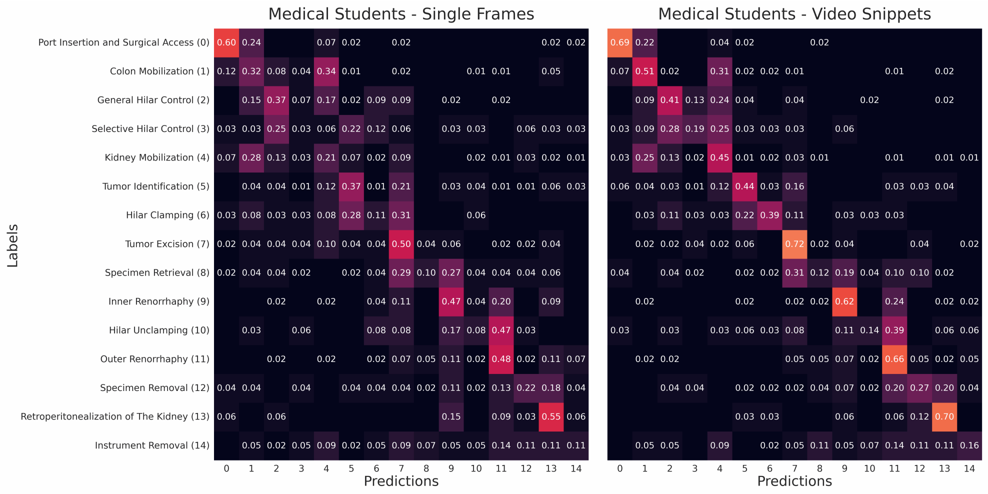

(a) Medical students Performance

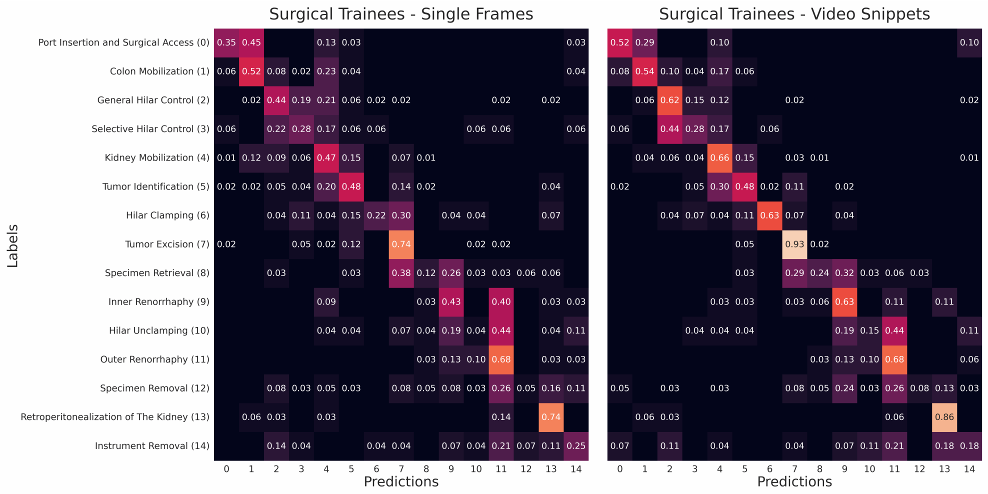

(b) Surgical Trainees Performance

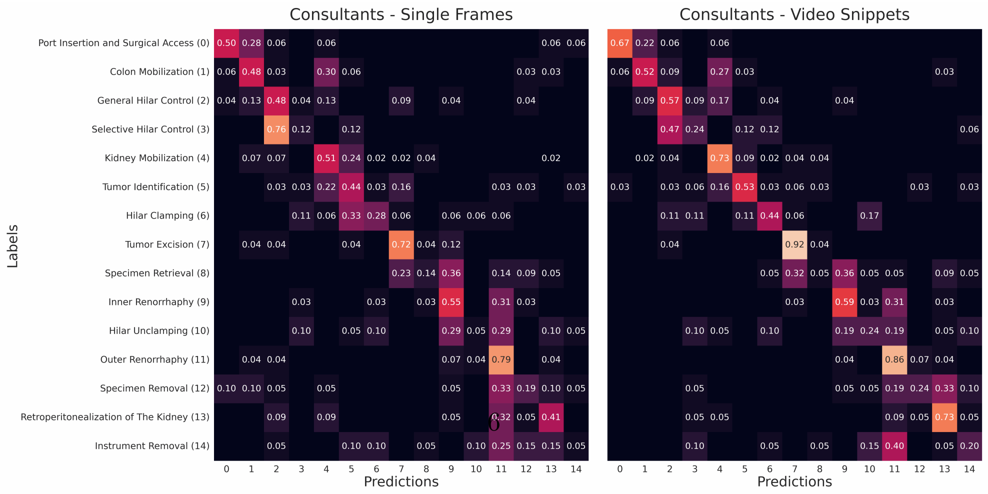

(c) Consultants Performance

Fig. S3: Confusion matrices of survey participants normalised over the predictions

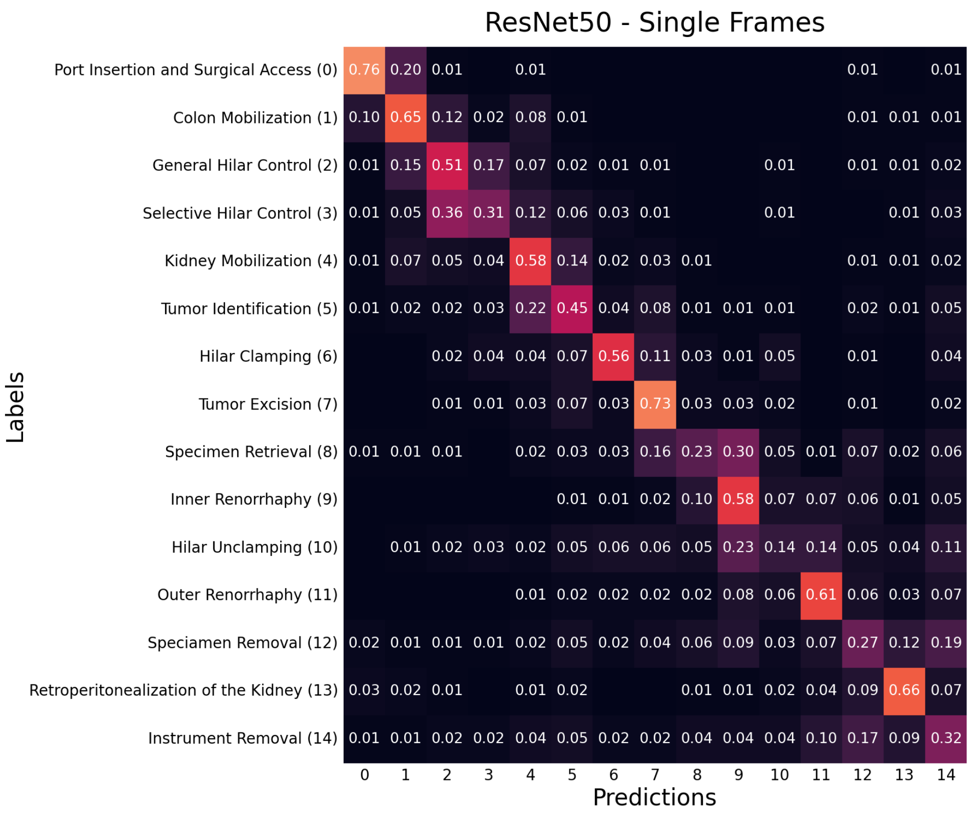

(a) ResNet50 performance for single frames classification

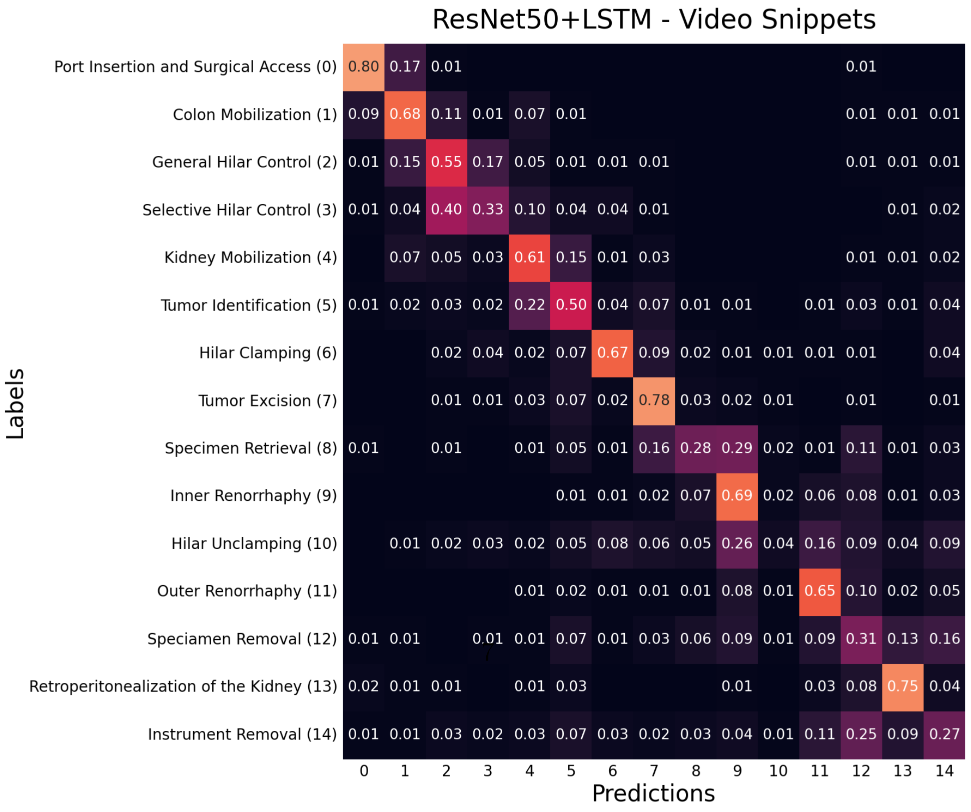

(b) ResNet50+LSTM performance for 10-seconds video snippets classification

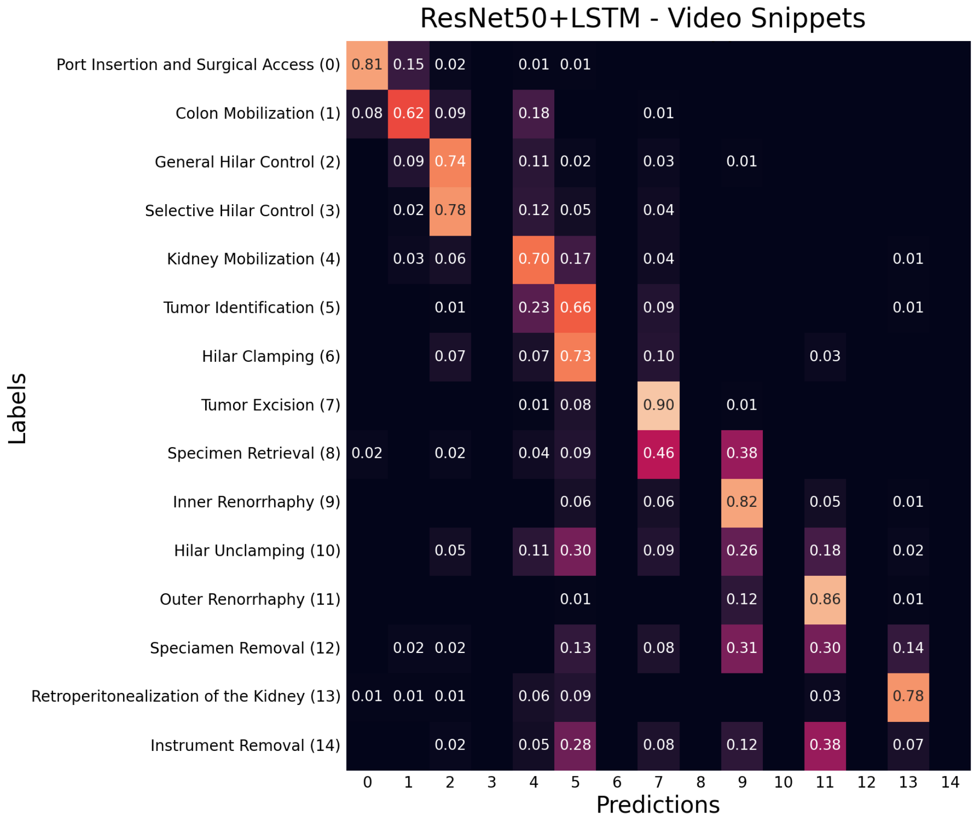

(c) ResNet50+LSTM performance for 60-seconds video snippets classification

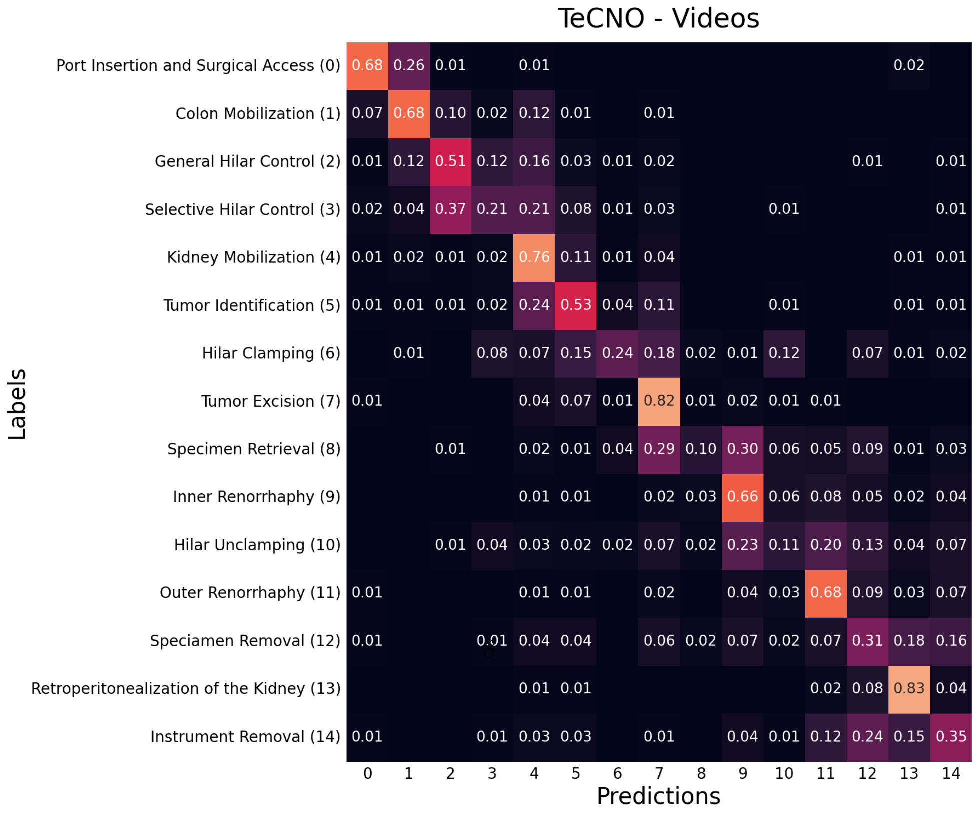

(d) TeCNO performance on phase classification

Fig. S4: Confusion matrices normalised over the predictions of AI models trained over 100% RAPN dataset.

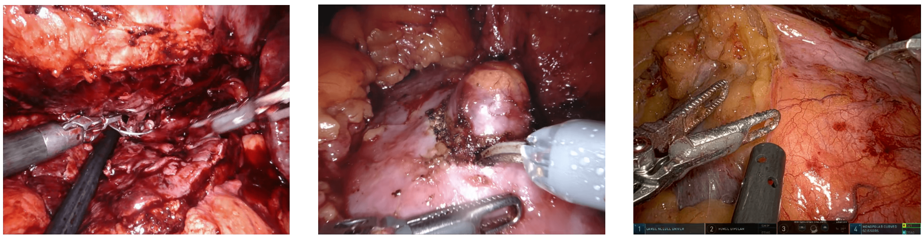

(a) Clear single frames examples.

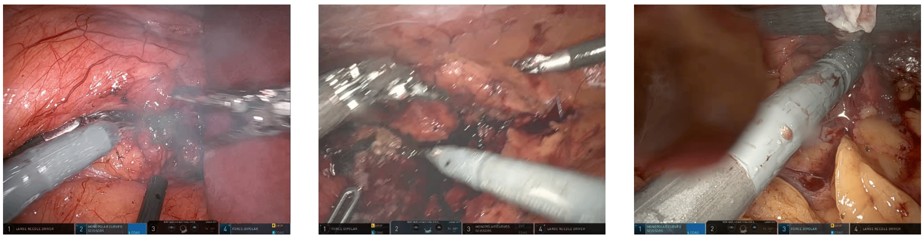

(b) Difficult single frames examples.

Fig. S5: Single Frames sampled from the set of questions proposed to human raters.
